# A straightforward framework to harmonize computational pathology

**DOI:** 10.1016/j.jpi.2026.100676

**Published:** 2026-05-11

**Authors:** Amanda Dy, Jochen K. Lennerz

**Affiliations:** a Electrical, Computer, and Biomedical Engineering, Toronto Metropolitan University, Toronto, ON, Canada; b Natera, Austin, TX, USA

**Keywords:** DICOM, Standard, Biomarker, Foundation model

## Abstract

Computational pathology datasets are commonly described using inconsistent and locally defined terminology, often conflating biological units (patients, specimens), lab preparations (blocks, slides), and derived digital data (whole-slide images (WSIs), tiles, and patches). This ambiguity complicates interpretation of training data composition, limits comparability across studies, and affects claims regarding dataset scale, generalizability, and clinical relevance. We reviewed widely cited foundation models and observed substantial variability in how disease representation is reported, highlighting the need for a standardized hierarchical framework. We propose a straightforward harmonization framework by aligning dataset descriptions with the hierarchical information model defined by DICOM. The framework distinguishes clinical, lab, and digital domains and recommends explicit reporting across hierarchical levels. Adoption of this terminology enables transparent dataset characterization, improves reproducibility, and facilitates regulatory and clinical translation of computational pathology. This pragmatic framework provides an immediately implementable pathway towards harmonized reporting in artificial intelligence-driven pathology research.

Artificial intelligence (AI) is rapidly transforming medicine, and pathology is no exception. The emergence of computational pathology, enabled by the adoption of digital pathology, represents a fundamental transition in how histopathology data are generated and analyzed. High-resolution digitization of glass slides introduces what is arguably a major missing diagnostic modality into the digital medical record, making these data amenable to large-scale computational analysis.

Over the past decade, the field has witnessed the development of foundation models trained on massive collections of histopathology images.^8^ These models promise accelerated biomarker discovery, automate diagnostic tasks, and enable new clinical decision-support tools. However, a key prerequisite for evaluating such models is understanding what types of biological material and disease indications are represented in the underlying training data.

During AI model development, we noticed a recurring source of confusion: computational pathology studies frequently report datasets using locally defined terminologies (for example, *samples*, *images*, or *cases*). These terms often conflate fundamentally different hierarchical levels of pathology data. To illustrate this conflation, we reviewed several widely cited pathology foundation models,^1^, ^2^, ^4^, ^6^, ^7^, ^9^, ^11^, ^12^ and how the indication “colorectal cancer” was represented in their training datasets. The results, summarized in Table 1, reveal substantial variability in how this indication is described and quantified. Notably, only some of the recent studies report detailed disease representation across more than one analytical level (for example, *specimen-, slide-, or dataset-level counts*). Hierarchical reporting of the important elements is currently not standardized. The current opportunity is therefore not to replace existing practice, but to harmonize these efforts into a clear and interpretable standard. In many cases, the terminology used makes it difficult to determine the true biological representation within the training data. This ambiguity is not trivial. Foundation model comparisons and benchmarks, dataset sizes, generalization evidence, and performance claims depend heavily on the level at which data are counted and interpreted.

**Table 1:** Foundation model summary—attempt to understand the representation of the indication “colorectal cancer.”^a^

Foundation model	Training data scale	Description of colorectal*/gastrointestinal* representation
UNI^1^	100 M tiles from 100 k WSIs	Colon not explicitly listed; bowel tissue accounts for 8% of WSIs
Virchow^9^	2B tiles from 1.5 M WSIs	Colon accounts for ∼3.2% of dataset; ∼12% of specimens
Virchow2^13^	3.1B tiles from 1 M WSIs	Colon accounts for ∼7% of WSIs
Phikon^3^	43 M patches from 6 k WSIs	Colon accounts for ∼10% of WSIs (derived from TCGA-COAD, TCGA-READ)
Phikon2^4^	460 M tiles from 200 k WSIs	Colorectal accounts for ∼9% of WSIs
Prov-GigaPath^11^	1.3B tiles from 171 k WSIs	Colon not explicitly listed; bowel tissue accounts for 30% of WSIs and 23% of patients
HIBOU-L^7^	1.2B tiles from 1.1 M WSIs	Gastrointestinal accounts for 31% of WSIs
HIBOU-B^7^	512 M tiles from 1.1 M WSIs	Gastrointestinal accounts for 31% of WSIs
CTransPath^10^	15 M patches from 30 k WSIs	Colon not explicitly listed; TCGA lists >25 anatomic sites potentially including TCGA-COAD and/or TCGA-READ
PLUTO^6^	1.5 M tiles from 159 k WSIs	Lower gastrointestinal accounts for ∼12% of WSIs
RudolfV^2^	1.2B patches from 134 k WSIs	Lower gastrointestinal accounts for ∼7% of WSIs
PathOrchestra^12^	140 M patches from 288 k WSIs	Colon accounts for ∼1.4% of WSIs

a Abbreviations: B, billion; COAD, colon adenocarcinoma; k, thousand; M, million; READ, rectal adenocarcinoma; TCGA, The Cancer Genome Atlast; WSI, whole slide image.

Most AI articles inadvertently mix these layers. For example, “samples” may refer to *patients* or *slides*, and “images” may refer to *slides* or *patches*. Simply put, the applied terminology is often local jargon and represents an unintentional source of ambiguity. The key observation is that many AI teams work at different abstraction levels within the pathology data hierarchy. Some studies operate at the *patient- or specimen-level*, others at the *slide-level*, and many foundation model approaches operate at the *tile- or patch-level*. As illustrated in Table 1, the absence of clear and universally adopted terminology forces teams to rely on locally defined hierarchies, complicating comparisons across studies.

Based on prior work describing digital pathology information models,^5^ we propose a simple and practical call to action. Aligning terminology with the DICOM information model provides a transparent and interoperable framework for describing *patient*-, *specimen*-, *slide*-, and *image*-level data in AI studies. However, a practical explanation of how this framework can be applied to AI dataset reporting is currently missing.

As a starting point, we distinguish between three domains that are frequently conflated (Table 2). A straightforward solution is to align the local pathology or computational terminology with the established hierarchy defined by DICOM (Table 3; Fig. 1). To enable adoption, we provide three components as a conceptual alignment of the proposed solution.

**Table 2:** Three domains in computational pathology.

Domain	Example hierarchy
Clinical/specimen hierarchy	Patient → Case → Specimen
Lab preparation	Specimen → Block → Slide → Stained slide
Digital imaging	Scan → WSI → Levels → Tiles → Patches

**Table 3:** Practical harmonization framework for computational pathology reporting.

Conceptual level	DICOM term	Commonly used pathology terms	Commonly used AI/ML terms
Individual	Patient	Patient	Patient
Clinical episode	Study	Case, accession(ing)	Case
Tissue acquisition	Specimen	Specimen, sample, tissue sample, container^a^	Specimen
Tissue subdivision	Specimen component	Part, tissue piece	Sample
FFPE block	Container^a^	Block, cassette	Block
Glass slide	Specimen preparation step	Slide	Slide
Histochemical process	Stain	H&E, IHC, special stain	H&E, IHC, stain
Image acquisition	Series*/*Acquisition	Scan	Scan, digitization
Digital slide	Image*/*Frame set	WSI, digital slide	WSI
Image subdivision	Frame*/*Region	Tile	Tile, region
Model input	Derived object	Patch, crop	Patch, tile

*Abbreviations*: AI, artificial intelligence; FFPE, formalin fixed paraffin-embedded; H&E, hematoxylin & eosin stain; IHC, immunohistochemical stain; ML, machine learning; WSI, whole-slide image/imaging.

a In some pathology labs, the term *container* may refer to the wet-lab term “specimen jar,” e.g., the “container” received from surgery. This may cause confusion with the term c*ontainer* in the DICOM model, where the term is used in a broader information-model sense to denote a physical holder or organizational object that contains or groups specimens, preparations, or related data elements within a standardized hierarchy.

**Fig. 1:**
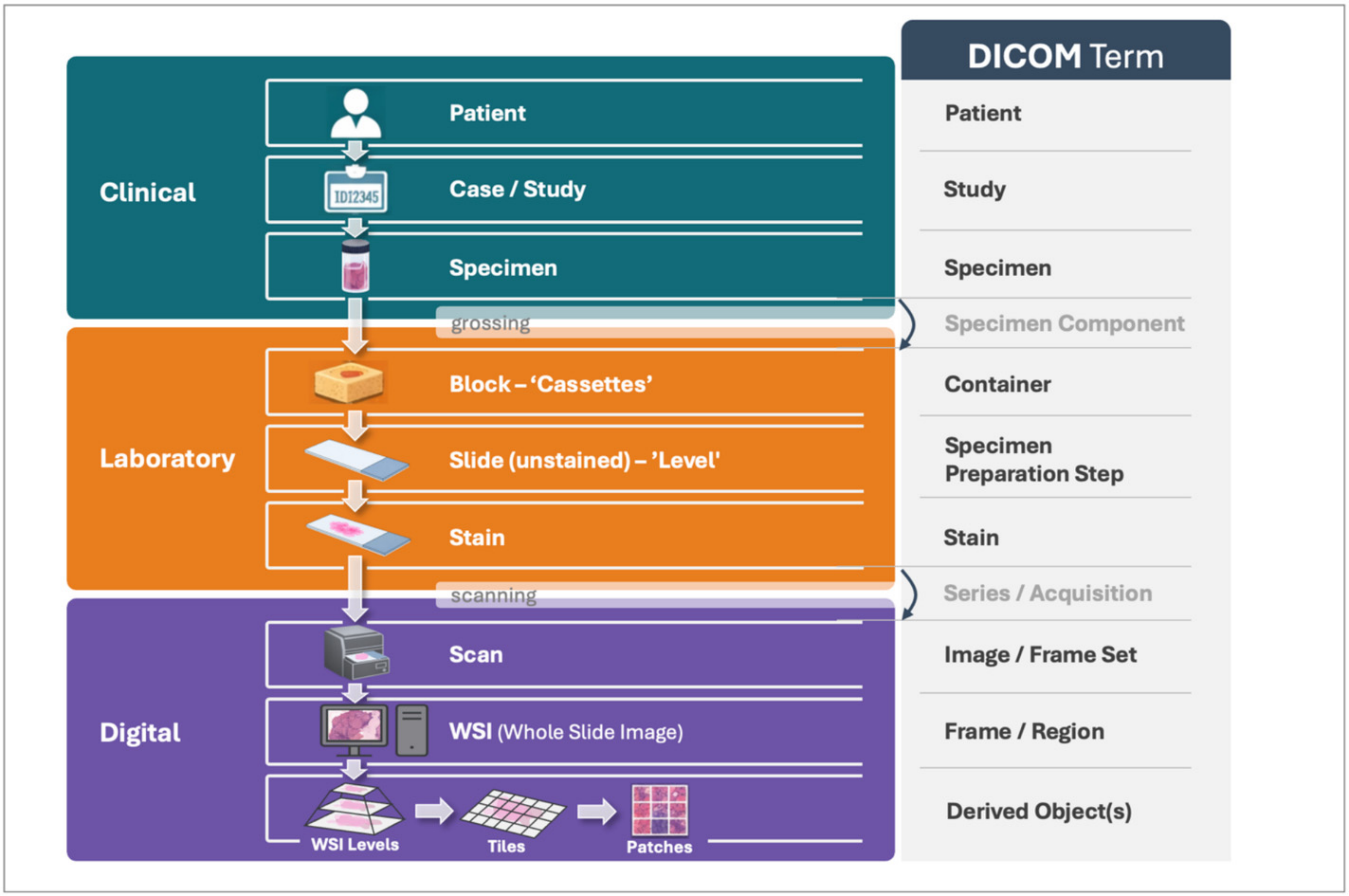
**Conceptual mapping of the histopathology workflow to DICOM terminology.** The diagram illustrates the progression from clinical context through lab processing to digital image generation, alongside corresponding DICOM terms. Clinical entities include patient, case/study, and specimen. During lab processing, specimens are grossed into blocks (cassettes), sectioned into slides (levels), and stained. Digital acquisition begins with slide scanning, producing WSIs, which may be represented at multiple levels and further subdivided into tiles and patches. The right panel lists corresponding DICOM concepts, linking clinical, lab, and digital objects across the end-to-end workflow.

First, Fig. 1 proposes a visual outline of the histopathology workflow mapped to DICOM terminology to clearly state which level represents the primary analytical unit. We recommend making this explicit in dataset descriptions. Importantly, we point out that *grossing* and *scanning* mark the transitions between the different stages of tissue processing that must be represented in DICOM when striving for complete provenance.

Second, we propose that authors should provide counts, reported separately, at the *patient*, *specimen*, *block*, *slide*, *WSI*, and *patch* levels to clarify the biological units represented and the derived image units used for model development. The terms *tiles* and *patches* are frequently used interchangeably in computational pathology. For clarity, *tiles* generally refer to systematically generated fixed-size image regions extracted from a predefined grid, whereas *patches* more broadly include any cropped image region selected for analysis, whether grid-based, randomly sampled, or annotation-guided. Notably, in many WSI file formats, *tiles* additionally refer to internal image blocks used for storage, compression, and access. These file format *tiles* generally do not correspond to the analysis *tiles* or *patches* used for model development. Because usage varies across studies, authors should explicitly state how image regions were generated, selected, and processed rather than relying on terminology alone.

Here, we share what a standardized dataset description might look like: “*The dataset included 1250 patients with colorectal cancer, contributing 1620 surgical specimens, 2950 FFPE blocks, 4300 H&E slides, and 4300 WSIs. From these WSIs, 210 million non-overlapping tumor containing image patches were extracted at Level 0 at 20× magnification. Each patch measured 256 × 256 pixels at 0.5 microns-per-pixel.”*

At present, dataset descriptions at this level of granularity remain the exception rather than the norm in computational pathology reports. To illustrate how this framework can be operationalized in routine manuscript preparation, Box 1 presents four example standardized dataset descriptions that authors may adapt to different study settings: (1) a general reporting template, (2) a small biopsy cohort, (3) a multi-modal foundation model, and (4) a multi-institutional foundation model dataset.

**Box 1:**
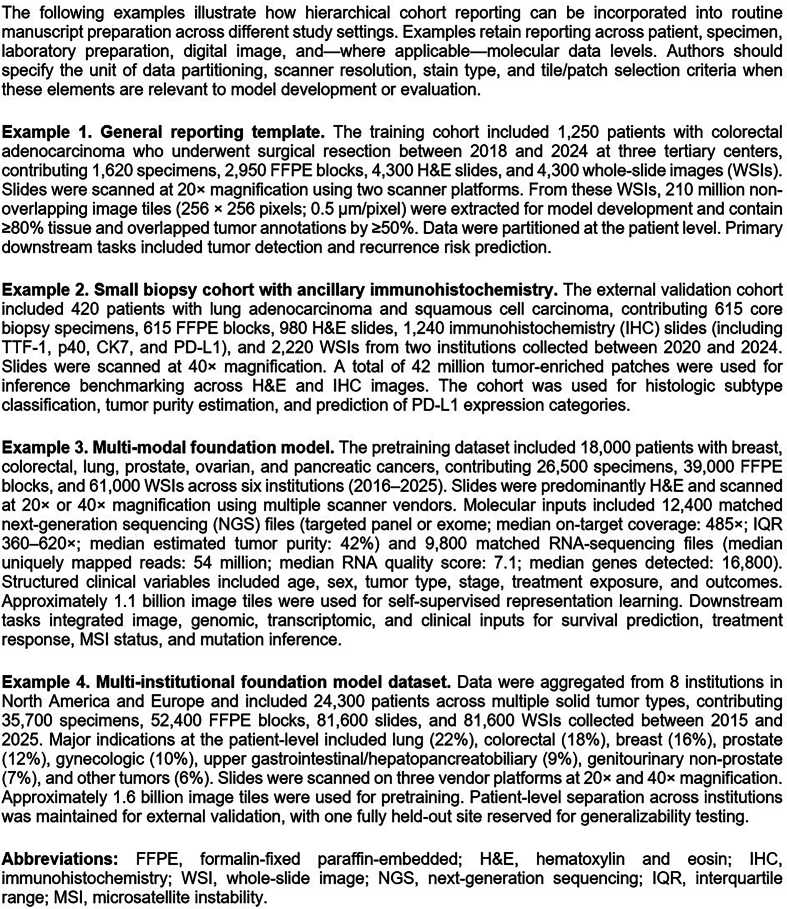
Example standardized dataset descriptions incorporating recommended minimum metadata.

Third, the proposed framework facilitates three essential questions: Which biological units are represented (patients, specimens)? Which lab preparations were generated (blocks, slides)? Which digital data were used for AI training (WSIs, tiles, and patches)? To facilitate immediate implementation, we propose a concise reporting checklist (Table 4) that can be integrated into existing reporting norms by authors, reviewers, journals, benchmark consortia, and dataset repositories to promote transparent and harmonized cohort descriptions.

**Table 4:** Practical checklist for transparent dataset reporting in computational pathology.

Item	Reported	Content examples
Number of patients	☐	*n* = patients
Number of cases	☐	*n* = cases
Number of specimens or part types	☐	*n* = specimen
Number of blocks*/*cassettes	☐	*n* = blocks
Number of slides	☐	*n* = slides
Number of WSIs	☐	*n* = WSI (rescans)
Magnification	☐	20× vs. 40×^a^
Number of tiles*/*patches	☐	*n* = tiles*/*patches
Tile size*/*patch size	☐	Pixel × pixel
Tile*/*patch sampling strategy	☐	% overlap, % tumor area
Pixel resolution	☐	Microns-per-pixel
Dataset-level split used^b^	☐	Train 70: tune 10: test 20
Institutions	☐	*n* = site count
Tumor types*/*Diseases	☐	N and list of indications
Scanner vendors*/*models	☐	Vendor, Model, City, Country
Stain type	☐	H&E, IHC, Ki-67, PD-L1, etc.
Special conditions	☐	e.g., missing levels explained

Recommended reporting elements spanning biological units, lab preparations, and derived digital data. The checklist is intended as a pragmatic aid rather than a mandatory standard and may be adapted to the scope of individual studies.

Abbreviations: H&E, hematoxylin and eosin stain; IHC, immunohistochemistry; WSI, whole-slide image.

a 20×/40× denotes optical lens designations from conventional microscopes and image resolution is more accurately described by pixel resolution.

b e.g., for model development.

Beyond conceptual alignment, clear hierarchical terminology also extends beyond imaging modalities, for example, to capture annotations, or research-related metadata. Dataset descriptions using the hierarchical information model defined by DICOM provide a practical pathway towards harmonization. For example, modern molecular workflows may start from different levels of the pathology hierarchy. Specifically, nucleic acids may be extracted from the *block*, separate *tissue sections*, or digitally defined *sub-regions* but are used to render decisions at the *patient-level*. Importantly, the proposed framework can thereby be understood as a pragmatic reporting aid rather than a rigid standard.

The proposed DICOM terminology is not without limitations. First, some DICOM terms do not fully reflect routine lab language. For example, in wet-lab pathology, the term *container* commonly refers to the *specimen jar* or transport vessel, whereas within the DICOM hierarchy *container* refers to the downstream physical or information object such as the *paraffin block* context. Because this dual use of the term can create persistent ambiguity in pathology workflows, a practical explanation is provided in Box 2. To minimize ambiguity, implementations should pair standard DICOM terms with familiar operational labels such as *specimen jar*, *cassette*, *FFPE block*, or *slide*. Second, existing shared and real-world datasets are often incomplete with respect to metadata across hierarchical levels, meaning that many studies may be unable to report all elements of the proposed schema retrospectively. Nonetheless, even partial adoption of a transparent hierarchy would substantially improve the interpretability and comparability of computational pathology studies. Third, although DICOM terms can clarify the point of origin of data within the pathology workflow, they do not determine their biological, clinical, or analytical meaning. For example, the exact representation of mucinous non-small cell lung cancer at the patch level in a pretrained multimodal foundation model can currently not easily be determined. Large numbers of derived image units should not be overinterpreted as equivalent to large numbers of independent biological samples, because many tiles or patches can originate from comparatively few samples.

**Box 2:**
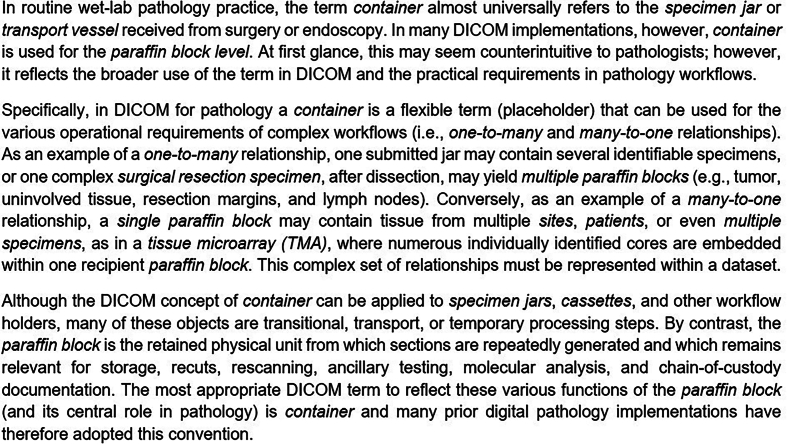
The DICOM term c*ontainer.*

Computational pathology is entering an era of increasingly large datasets and foundation models trained on billions of image patches. Yet, the terminology used to describe these datasets remains inconsistent across studies. A shared hierarchical reporting framework will improve transparency, reproducibility, regulatory evaluation, and clinical translation in AI-based pathology.

## Funding

AD is funded by the Natural Sciences and Engineering Research Council of Canada (NSERC) Vanier Canada Graduate Scholarship (198875).

## Declaration of competing interest

JKL is employed by Natera, Inc., Austin, TX, USA.

The authors declare that they have no known competing financial interests or personal relationships that could have appeared to influence the work reported in this article.

## References

[1] Chen R.J., Ding T., Lu M.Y. (2024). Towards a general-purpose foundation model for computational pathology. Nat Med.

[2] Dippel J., Feulner B., Winterhoff T. (2024).

[3] Filiot A., Ghermi R., Olivier A. (2023). Scaling self-supervised learning for histopathology with masked image modeling. medRxiv.

[4] Filiot A., Jacob P., Kain A.M., Saillard C. (2024).

[5] Herrmann M.D., Clunie D.A., Fedorov A. (2018). Implementing the DICOM standard for digital pathology. J Pathol Inform.

[6] Juyal D., Padigela H., Shah C. (2024).

[7] Nechaev D., Pchelnikov A., Ivanova E. (2024).

[8] Rashidi H.H., Pantanowitz J., Chamanzar A. (2025). Generative artificial intelligence in pathology and medicine: a deeper dive. Mod Pathol.

[9] Vorontsov E., Bozkurt A., Casson A. (2024). A foundation model for clinical-grade computational pathology and rare cancers detection. Nat Med.

[10] Wang X., Yang S., Zhang Jun (2022). Transformer-based unsupervised contrastive learning for histopathological image classification. Med Image Anal.

[11] Xu H., Usuyama N., Bagga J. (2024). A whole-slide foundation model for digital pathology from real-world data. Nature.

[12] Yan F., Wu J., Li Jiawen (2025). PathOrchestra: a comprehensive foundation model for computational pathology with over 100 diverse clinical-grade tasks. Npj Digit Med.

[13] Zimmermann E., Vorontsov E., Viret J. (2024).

